# Molecular and morphological signatures of drought and salinity stress in *Olea europaea*

**DOI:** 10.3389/fpls.2026.1813434

**Published:** 2026-04-14

**Authors:** Gaia Salvatore Falconieri, Michael Bianchi, Laura Bertini, Anna Rita Taddei, Carmen Morales-Rodríguez, Carla Caruso, Andrea Vannini, Cristian Silvestri, Silvia Proietti

**Affiliations:** 1 Department of Ecological and Biological Sciences, University of Tuscia, Viterbo, Italy; 2 Center of Large Equipments, Section of Electron Microscopy, University of Tuscia, Viterbo, Italy; 3 Department for Innovation in Biological, Agrofood and Forest Systems, University of Tuscia, Viterbo, Italy; 4 Institute for Sustainable Plant Protection, National Research Council of Italy, Torino, Italy; 5 Department of Agriculture and Forest Sciences, University of Tuscia, Viterbo, Italy

**Keywords:** drought, *Olea europaea* cv. Canino, oxidative stress, plant growth, plant pigments, salinity

## Abstract

**Introduction:**

Abiotic stresses such as drought and salinity represent major constraints to plant productivity and threaten the cultivation of economically important species such as *Olea europaea*. In this study, we characterized the response of *O. europaea* cv. Canino to drought and salinity using a multidisciplinary approach. *In vitro*–grown olive plants were exposed to drought and salinity stress and analyzed at morphological, molecular, and biochemical levels.

**Methods:**

Plant growth and leaf trichome density were examined by electron microscopy, oxidative damage was assessed by confocal microscopy and biochemical assays, and the expression of key stress-responsive genes was quantified by quantitative RealTime PCR (qRT-PCR). Photosynthetic pigments, carotenoids, and anthocyanins were also quantified to evaluate stress-related changes in energy metabolism.

**Results and discussion:**

Both stresses significantly impaired plant growth and modified leaf trichome morphology and density. Stress treatments also altered the levels of photosynthetic pigments, carotenoids, and anthocyanins, suggesting a reduction in photosynthetic efficiency. Moreover, drought and salinity induced oxidative stress, although to different extent. Together, these observations demonstrated that olive cv. Canino employs coordinated morphological, physiological, and molecular strategies to respond to the adverse effect of drought and salinity. Overall, our findings identify key morphological and molecular signatures associated with drought and salinity responses in olive cv. Canino, providing insights that could support its cultivation in areas affected by these stresses.

## 1 Introduction

Climate change is increasingly affecting crop productivity worldwide, with negative impact on human health, socio-economic systems, agriculture and water resources (Hoegh-Guldberg et al., 2018; IPCC, 2023). An emblematic aspect of climate change is represented by global warming, leading to an increased frequency and severity of several biotic and abiotic stresses that negatively harm agriculture globally (Yuan et al., 2024). Among the abiotic stresses, drought and salinity represent the most crucial ones limiting crop productivity, particularly in the Mediterranean and other semi-arid regions (Ben Ahmed et al., 2009; Hoegh-Guldberg et al., 2018; Majikumna et al., 2024). The Mediterranean basin appears to be particularly vulnerable to the effects of climate change (Majikumna et al., 2024). In fact, this region is characterized by dry summers and mild and wet winters, and in recent decades it experienced water scarcity, groundwater salinization and other environmental stresses (Urdiales-Flores et al., 2023; Lazoglou et al., 2024). Drought and salinity are definitely expected to increase significantly in the coming years (Jalil and Ansari, 2020; Sharma et al., 2022).

Drought is due to water scarcity or to excessive evapotranspiration, while salinity is caused by an increase in salt concentration in the soil, especially inorganic ions (Na^+^ and Cl^-^) (Morsy et al., 2020). Drought and salinity, as well as most of the abiotic stresses, represent complex environmental challenges that affect multiple facets of plant biology, influencing morphological, physiological, biochemical and molecular traits (Salehi-Lisar and Bakhshayeshan-Agdam, 2016; Mustapha and Zineddine, 2024). At a morphological level, drought leads to a reduction in shoots growth and faster fruit ripening (Sharma et al., 2022), while under salinity stress, chlorosis and necrosis of leaves are the most evident traits (Ledesma et al., 2016). Some of the major effects triggered by either drought and salinity are the decrease of CO_2_ availability caused by limited diffusion through the stomata and the mesophyll (Flexas et al., 2007; Haghighi et al., 2022) or altered photosynthetic metabolism (Karami et al., 2025). In addition, drought and salinity stresses disrupt cell membrane integrity, leading to lipid peroxidation and protein degradation. These stresses also disturb redox homeostasis by promoting the accumulation of reactive oxygen species (ROS), which further exacerbates cellular damage and impairs physiological functions (Abdallah et al., 2018). To counteract high ROS levels, plants have developed complex defense mechanisms, including enzymatic and non-enzymatic antioxidant systems, making ROS scavengers as valuable markers of oxidative stress experienced under both stress conditions (Foyer and Noctor, 2005).

Olive (*Olea europaea* L.) is one of the most iconic crops of the Mediterranean region, accounting for approximately 98% of global olive oil production, which is highly appreciated worldwide (Fabbri, 2023). It belongs to the Oleaceae family, and it is a medium-sized, evergreen, long-living and thermophilic tree, with a behavior that varies from glycophytic to halophilic, depending on genotype (Sodini et al., 2023). It is the only member of the Oleaceae family to be cultivated for its edible fruits from which olive oil, a healthy food with antioxidant and anti-inflammatory properties, is produced. The olive tree is well-known for its wide range of adaptation mechanisms to cope with Mediterranean climate and abiotic stress cues like drought and salinity (Faraloni et al., 2011; Araújo et al., 2021; Tadić et al., 2021, 2024; Trabelsi et al., 2019, 2022; Salimonti et al., 2025). Olive copes with drought through coordinated physiological, anatomical, and metabolic responses. These include osmotic adjustment through the accumulation of compatible solutes such as proline, sugars, and sugar alcohols, structural adaptations that reduce water loss, and the activation of antioxidant defense systems to control drought-induced ROS accumulation. In addition, drought stress modulates primary and secondary metabolism, promoting the accumulation of carbohydrates and phenolic compounds that contribute to osmotic balance, energy supply, and protection against oxidative damage (Parri et al., 2024). Analogously, olive plants experiencing salinity stress reduced photosynthesis and chlorophyll content due to stomatal closure and impaired chloroplast function. Salt exposure also disrupts nutrient uptake and causes oxidative stress through excessive ROS accumulation. To counteract this, olives activate antioxidant enzymes and accumulate compatible solutes such as proline and glycine betaine to maintain osmotic balance and protect cellular structures (Hussain et al., 2026). Interestingly, at molecular level, several studies have highlighted specific genes, among others those involved in oxidative stress, such as *ABA1* (Benny et al., 2020)*,AREB3* (Benny et al., 2020), *CHS* (Rossi et al., 2016), *OeFAD2-2* (Moretti et al., 2019) and *ProDH* (Bazakos et al., 2015), photosynthesis, such as *CA-β2* (Perez-Martin et al., 2014), ion transport, such as *NHX* (Rossi et al., 2016), cellular metabolic process, such as HMG (Bazakos et al., 2015), as molecular markers for drought/salinity stress in several olive cultivars.

To date, there is limited information on the morphological, physiological and biochemical features perturbed in *Olea europaea* cv. Canino in response to salinity (Bashir et al., 2021) and to the best of our knowledge, none of the published work considered to investigate drought stress. In the present work, we aim to provide new insights into the response of the olive cv. Canino *in vitro* grown to drought and salinity stresses, describing how they affect plant growth, oxidative damage and photosynthetic pigments and the expression of key stress marker genes. *In vitro* cultures are extensively utilized as rapid and cost-effective screening platforms to evaluate plant tolerance to various abiotic stresses, such as drought, salinity, or osmotic stress, under controlled laboratory conditions (Wijerathna-Yapa and Hiti-Bandaralage, 2023). We have found that both stresses impacted plant growth, and morphology and density of olive leaves trichomes. At molecular level, eight genes such as *ABA1*, *AREB3*, *CA-β2*, *CHS*, *HMG*, *NHX*, *OeFAD2.2* and *ProDH* have been tested and validated by qRT-PCR as valuable marker genes of drought and/or salinity stress responses. Then, photosynthetic pigment concentration, carotenoids and anthocyanins were significantly perturbed by both salinity and drought. Lastly, drought and salinity induced oxidative stress, although to different extent, as shown by confocal microscope observation and biochemical markers analysis. Taken together, our results contributed to unraveling the morphological and molecular signatures of salinity and drought stress responses in olive cv. Canino.

## 2 Materials and methods

### 2.1 Plant material and growth conditions

The shoot cultures derived from *in vitro* propagation of olive cultivar Caninowere grown in 250 mL jar containing OM medium (Rugini, 1984) and 36 g/L mannitol, 2.2 g/L L-glutamine, 1 mg/L zeatin (added after filter sterilization), plus 0.6% plant agar (Duchefa, NL), for a period of four weeks. The pH of the medium was adjusted to 5.8 before autoclaving. Afterwards, four-week-old single-node explants were sub-cultured in jars, containing 50 mL of the growth medium described above. The medium was supplemented with either three different concentrations of NaCl (50, 100, and 200 mM) or with varying concentrations of polyethylene glycol (PEG) 4000 (1, 2, and 4%). The PEG concentrations were selected based on previous experiments to avoid lethal doses (Verslues et al., 2006; Wijerathna-Yapa and Hiti-Bandaralage, 2023). Representative photos of *in vitro* grown cultures are reported in **Supplementary Figure 1**. In each independent experiment, for each stress condition (NaCl 50 mM, 100 mM, 200 mM; PEG 1%, 2%, 4%) plus control, a total of three jars, each containing 10 explants, were settled. A total of three independent experiments was performed. The explants were kept in a growth chamber for four weeks under controlled conditions with a day/night cycle of 16/8 hours at an air temperature of 24 ± 1 °C, 80% relative humidity, and light intensity of 40 µmol m^-2^ s^-1^. All analyses were performed after the abovementioned four-week growth period, when the explants had developed 2–4 leaves.

### 2.2 Morphometric analysis

To evaluate shoot proliferation, the data on node number, shoot number, shoot length (cm), and internode length (cm) were collected. In each independent experiment, the final value of each parameter was calculated as the average of six independent measurements representing the mean of five randomly selected explants. The relative growth rate (RGR) index was also calculated as described by Gatti et al. (2017). Due to the disruptive analysis, in each independent experiment, the final value of RGR was calculated as the average of two independent measurements representing the mean of three explants randomly selected.

### 2.3 Scanning Electron Microscope analysis

For Scanning Electron Microscope (SEM) analyses, samples from 9 leaves were first fixed with osmium tetroxide vapor for 1 hour at room temperature and immersed in an aldehyde fixative (2.5% (v/v) glutaraldehyde and 2% (v/v) paraformaldehyde in 0.1 M cacodylate buffer, pH 7.2) overnight at 4°C. After three washings for 20-minute at 4 °C in the same buffer, they were dehydrated in a graded ethanol series. Samples were dried by the critical point method using CO_2_ in a Balzers Union CPD 020. Then, samples were attached to aluminum stubs using carbon tape and sputter-coated with gold in a Balzers MED 010 unit. The observations were made using a JEOL JSM 6010LA electron microscope.

### 2.4 Total RNA extraction, cDNA synthesis and quantitative real-time PCR

In each independent experiment, total RNA was extracted from 100 mg of plant leaves randomlyselected from three explants, pooled together. For RNA isolation, the Nucleospin^®^ RNA Plant kit (Macherey–Nagel, Düren, Germany) was used, in accordance with the manufacturer’s instructions. Extracted RNA was treated twice with DNase of the same kit to remove genomic DNA contamination. RNA concentration was estimated by measuring absorbance at 260 nm, whereas the OD_260_/OD_280_ and OD_260_/OD_230_ nm absorption ratios were calculated to evaluate RNA quality and purity (spectrophotometer UV-30 SCAN, ONDA). RNA concentration values of all analyzed samples are reported in **Supplementary Table 1**. RNA integrity was also verified by agarose gel electrophoresis, whereas the absence of DNA contamination was tested using 100 ng of total RNA as a template in a PCR reaction using β-actin specific primers for amplification. Complementary DNA (cDNA) was synthesized using the ImProm-II RT reverse transcription system (Promega, Madison, WI, USA) starting from 1 μg of RNA as template and using the oligo-dT primer for first strand synthesis.

A bibliographic search was carried out to select potential molecular markers for drought and salt stress in olive, based on their known roles and reported changes in expression in response to the abiotic stresses investigated in this study. Zeaxanthin epoxidase (ABA1 or ZEP), ABA responsive element binding protein 3 (AREB3), Carbonic anhydrase β2 subunit (CA-β2), chalcone synthase (CHS), high mobility group homologous (HMG), Na^+^/H^+^ antiporter (NHX), microsomal oleate desaturase 2 (OeFAD2-2) and Proline dehydrogenase (ProDH) were selected. ZEP, AREB3 and CA-β2 primer pairs were designed using Primer3 software (https://primer3.ut.ee); the other primers were taken from literature, as reported below. An *in-silico* PCR was performed to verify the appropriateness of designed primers. Quantitative Real-Time PCR (qRT-PCR) reactions were performed in 96-well plates on a Bio-Rad CFX96 real-time PCR thermal cycler (Bio-Rad, Hercules, CA, USA), using the SYBR green detection system. The amplification program was as follows: 95°C for 5 s; 40 cycles at 95°C for 5 s, primers annealing and extension at 60°C for 45 s. Following amplification, the melting curves ranging from 70 to 95°C (with a constant increase of 0.5°C every 5 s) were evaluated in order to check the PCR specificity. Each assay included no-template controls (NTCs). The gene expression of selected genes was normalized against the reference gene β-Actin (β-Act) as suggested in Rossi et al. (2016). The relative gene expression was determined using the 2^−ΔΔCt^ method. Output data was processed using the CFX ManagerTM Software (Bio-Rad, Hercules, CA, USA). The primers used are listed in **Supplementary Material** (**Supplementary Table 2**).

### 2.5 Reactive oxygen species detection in *Olea europaea* leaves

In each independent experiment, reactive oxygen species (ROS) were detected in six leaf samples randomly collected from three explants (two leaves per explant). The analysis was performed following previously described methods with minor modifications (Proietti et al., 2023; Falconieri et al., 2024), Briefly, ROS production was detected by using 2′,7′-dichlorofluorescein diacetate (DCFH_2_-DA; Sigma-Aldrich), which is oxidized in highly green fluorescent dichlorofluorescein (DCF) when ROS are present. ROS were detected in 2 mm leaf sections. The half leaf section was incubated at room temperature in 20 μM DCFH_2_-DA in 10 mM Tris–HCl solution (pH 7.4) for 45 min in the dark. As a negative technical control, the other half was incubated in 10 mM Tris–HCl (pH 7.4) only, under the same conditions. After staining, samples were washed three times in 10 mM Tris–HCl (pH 7.4) for 10 min to remove the fluorophore excess and ultimately mounted on glass slides. Fluorescence was then observed under an LSM 710 confocal microscope (Zeiss Microscopy) with a Plan Neofluar 20/1.30 objective. Two laser excitation lines were used (488 nm for probe detection and 561 nm for chlorophyll autofluorescence). Quantification of green fluorescence in all tested conditions was performed with ImageJ, version 1.53s (NIH, Bethesda, MD, USA) as in Falconieri et al. (2024) and reported as the integrated density mean.

### 2.6 Thiobarbituric acid reactive substance measurement

In each independent experiment, thiobarbituric acid reactive substances (TBARS), an indicator of lipid peroxidation, were quantified using 400 mg of leaf tissue randomly collected from three explants and pooled prior to analysis. Following the methodology described in Proietti et al. (2021), leaf powder was resuspended in 3 mL of 0.1% TCA and vortexed until homogenized. Following a 10 min centrifugation at 15000 g, 400 μL of the supernatant (or 0.1% TCA for the blank) was added to either 1 mL of 0.5% thiobarbituric acid (TBA) in 20% TCA (+TBA solution) or 1 mL of 20% TCA (−TBA solution) (dilution factor 1:3.5). The samples were incubated at 80°C for 30 min before being cooled in ice. After 5 min of centrifugation at 15000 g, the absorbance was measured at 532 nm, which represents the maximal absorbance of the TBA–TBARS complex, and at 600 nm to allow for non-specific turbidity adjustment. The molar extinction coefficient (ε_μM_ = 0.155 μM^−1^ cm^−1^) of malondialdehyde (MDA), one of the primary products of membrane degradation, was used to determine TBARS equivalent (nmol mL^−1^) as follows:


$$\left[\text{A}/\epsilon_{\mu \text{M}}\text{MDA}\right]\;\times \text{ dilution factor}$$

Where:


$$\begin{array}{c} \text{A }=\;\left[\text{A}532\text{nm }\left(+\text{TBAsolution}\right)\;-\text{ A}600\text{nm }\left(+\text{TBAsolution}\right)\right]\\ \;–\;\left[\text{A}532\text{nm }\left(-\text{TBAsolution}\right)\;-\text{ A}600\text{nm }\left(-\text{TBAsolution}\right)\right] \end{array}$$

### 2.7 Assays on reactive oxygen species scavenging enzymes

In each independent experiment, the analysis was conducted on one gram of leaf samples randomly selected from six explants, pooled together. Powder was resuspended in 5 ml of a cold extraction solution containing 50 mM sodium phosphate buffer (pH 7.5), 1 mM EDTA, 1% (w/v) polyvinylpyrrolidone, 3 mM DTT, and a cocktail of protease inhibitors (Complete ULTRA tablets, Roche, Basel, Switzerland). The homogenate was centrifuged at 9000 g for 15 min at 4°C (Universal 32R, Hettich, Tuttlingen, Germany), and the supernatant was utilized for enzyme activity assays. According to Proietti et al. (2021), superoxide dismutase (SOD) and catalase (CAT) activities were measured. SOD activity was measured using the SOD determination kit (Sigma-Aldrich, Uppsala, Sweden). The SOD Assay Kit is based on the utilization of Dojindo’s water-soluble tetrazolium salt (WST) (2-(4-iodophenyl)-3-(4-nitrophenyl)-5-(2,4-disulfophenyl)-2H tetrazolium, monosodium salt), which is reduced by the superoxide anion to form a water-soluble formazan dye detectable at 440 nm. The rate of reduction of WST is linearly related to the activity of xanthine oxidase, which produces superoxide anion, and it is inhibited by SOD, which uses superoxide anion. Thus, the SOD activity can be determined by measuring the decrease of absorbance at 440 nm which is proportional to the SOD inhibitory activity corresponding to the quantity of total protein extract required to suppress formazan formation by 50% (IC_50_). CAT activity was determined by measuring the reduction in absorbance at 240 nm at 25°C caused by H_2_O_2_ decomposition (ε_mM_ = 0.0436 mM^−1^ cm^−1^). The reaction mixture (1 mL final volume) comprised 19 mM H_2_O_2_ in 50 mM potassium phosphate buffer (pH 7.0); the reaction was initiated by adding 50 μg of protein extract. The specific activity of CAT was measured in μmoles of hydrogen peroxide consumed per minute per mg of protein sample. Ascorbate peroxidase (APX) and guaiacol peroxidase (POD) activities were measured according to Bertini et al. (2019). The decrease in absorbance at 290 nm due to ascorbate oxidation was used to calculate APX activity (ε_mM_ = 2.8 mM^−1^ cm^−1^). The reaction buffer contained 50 mM of potassium phosphate, 0.5 mM of ascorbate, 0.1 mM of H_2_O_2_, and 0.1 mM of EDTA (pH 7.6). POD activity was measured at 30 °C in a reaction mixture containing 0.4% guaiacol (v/v) and 0.03% H_2_O_2_ in 100 mM potassium phosphate buffer (pH 7.0). The reaction was started by adding 2 mg of protein extract, and the increasing absorbance at 470 nm resulting from the formation of tetraguaiacol (ε_mM_ = 26.6 mM^–1^ cm^–1^) was monitored.

### 2.8 Determination of chlorophylls and carotenoids content

Determination of chlorophylls and carotenoids was conducted on 150 mg of leaf samples randomly selected from three explants, pooled together, according to Falconieri et al. (2024). Leaf powder was homogenized in liquid nitrogen and 1.5 ml of 80% acetone was added. The homogenate was centrifuged at 5314 g for 3 min, then the supernatant was recovered while the pellet was resuspended in 80% acetone (0.5 to 1 ml). The centrifugation and acetone suspension steps were repeated until the pellet was completely decolorized. The final supernatant volume of each sample was recorded. These data were used to calculate the concentration of chlorophylls and carotenoids. Spectrophotometer readings were taken at the following wavelengths using quartz cuvettes:

663 nm: preferential absorption of chlorophyll a
648 nm: preferential absorption of chlorophyll b
470 nm: preferential absorption of carotenoids

The concentration of chlorophylls and carotenoids was calculated as follows:


$$\text{Chl a }=\;12.25\;*\text{ AB}{\text{S}}_{663}–\;2.55\;*\text{ AB}{\text{S}}_{648}\left(µ\text{g}/\text{mL}\right)$$

$$\text{Chl b }=\;20.31\;*\text{ AB}{\text{S}}_{648}–\;4.91\;*\text{ AB}{\text{S}}_{663}\left(µ\text{g}/\text{mL}\right)$$

$$\text{Car }=\;\left(1000\;*\text{ AB}{\text{S}}_{470}–\;1.82\;*\;\left[\text{chl a}\right]\right)\;-\;\left(85.02\;*\;\left[\text{chl b}\right]\right)/198\;\left(µ\text{g}/\text{mL}\right)$$

Then, to obtain a concentration expressed in mg/g of fresh weight (FW), each value expressed in μg/mL was multiplied by the mL of the recovered supernatant. The resulting amount (in mg) was divided by the fresh weight (in mg).

### 2.9 Determination of anthocyanin content

Two-hundred mg of leaf samples randomly selected from three explants, pooled together, were used to determine anthocyanin content as described in Falconieri et al. (2024). Fresh leaf tissue was cold homogenized, followed by the addition of 2 mL of 0.1% HCl in methanol. At the end of the extraction, the supernatant was collected in Eppendorf tube and centrifuged at 10845 g for 10 min. A hundred μL of supernatant were added to 900 μL of 0.1% HCl in methanol in UV-Vis polystyrene cuvettes and absorption at 536 nm was evaluated. The blank contained 1 mL of 0.1% HCl in methanol only. The molar extinction coefficient of cyanidin-3-glucoside in the same solvent (ε_M_ = 30,400 mol^-1^ cm^-1^) was used to calculate the anthocyanin molar concentration.

### 2.10 Statistical analyses

All the analyses were performed by using a one-way analysis of variance and Tukey’s multiple comparisons test to assess the significant differences between the different treatments. Data distribution has been assessed by Shapiro-Wilk test and Kolmogorov-Smirnov test. All statistical analyses were performed with GraphPad Prism 10.1 (GraphPad Software Inc., San Diego, CA, USA). Descriptions of the sample size considered in each independent experiment have been reported specifically in each paragraph of the Materials and Methods. Each independent experiment was repeated three times.

## 3 Results and discussion

### 3.1 Effect of drought and salinity on growth performance of *Olea europaea* cv. Canino

**Figure 1:**
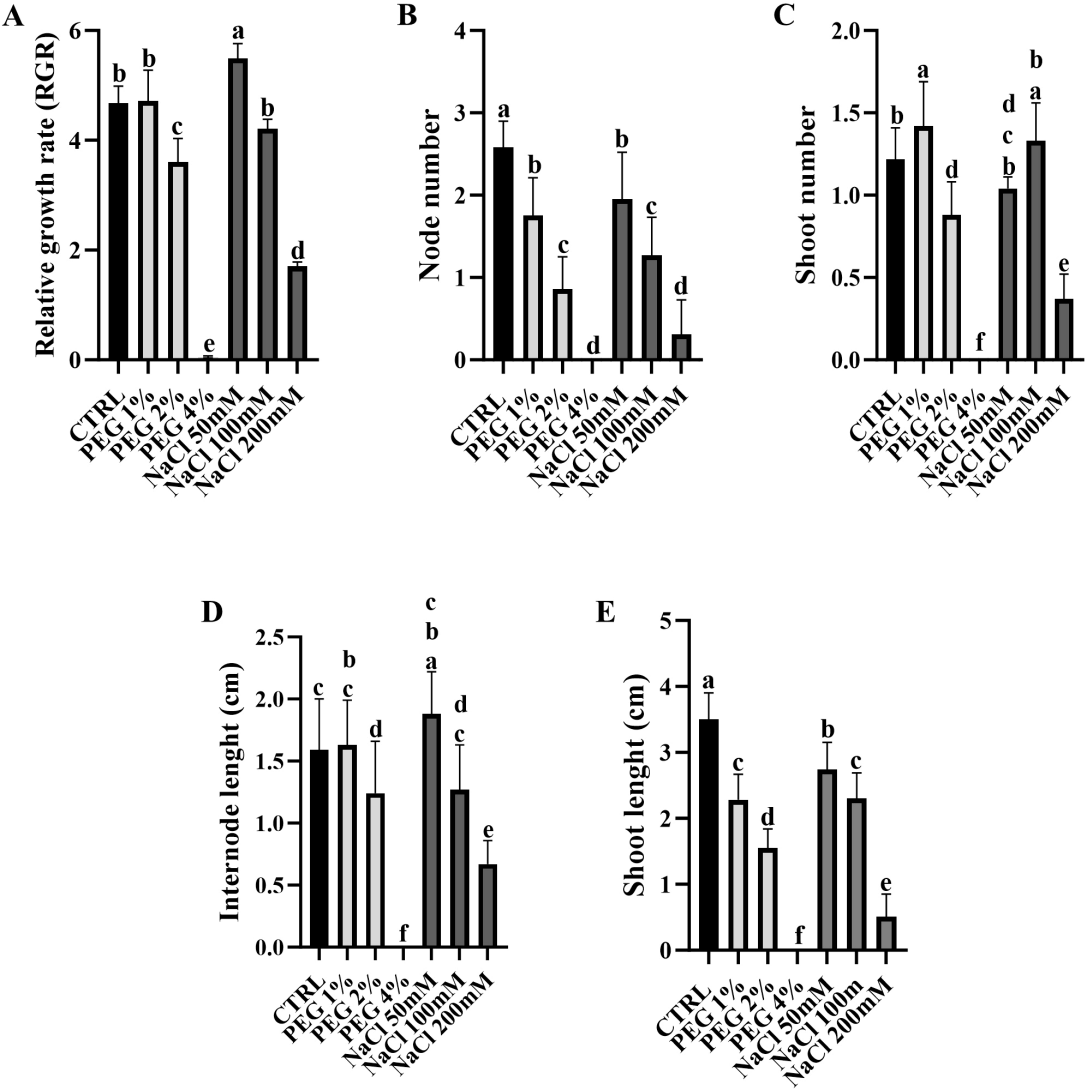
Morphometric parameters of *Olea europaea* plants under different concentrations of PEG and NaCl. Relative growth rate (RGR) **(A)**; Node number **(B)**; Shoot number **(C)**; Internode length **(D)**; Shoot length (cm) **(E)**. Letters above the bars indicate significant differences among the diverse treatments (One-way ANOVA; Tukey’s multiple comparison: p-value < 0.05).

Morphometric parameters are crucial indicators of plant health because they provide a sensitive and integrative measure of plant vigor, resource-use efficiency and stress response, especially in variable or harsh environments where olive trees typically grow (Gatti et al., 2017). Here, the effect of different PEG (mimicking drought) and NaCl (mimicking salinity) concentrations on relative growth rate (RGR), node and shoot number and on internode and shoot length were observed in olive plants (**Figure 1**).

In general, the increase in PEG and NaCl concentration leads to a decrease in all the analyzed parameters. Interestingly, the 4% PEG seems to be the worst condition experienced by the plants, in terms of growth parameters. Statistical analysis revealed a significant effect of NaCl and PEG concentrations, compared to control, in decreasing relative growth rate (RGR) of the cv. Canino, except for the lowest concentration (PEG 2% = 1.08, adj-p value = < 0.0001; PEG 4% = 4.63, adj-p value = < 0.0001; NaCl 50mM = -0.81, adj-p value = 0.0016; NaCl 200 mM = 2.98, adj-p value = < 0.0001). In particular, the highest RGR was recorded in shoots cultured on medium supplemented with 50 mM NaCl, as previously observed in Bashir et al., 2021. However, the cultivar experienced a notable decrease in RGR at higher NaCl (200 mM) and PEG (2% and 4%) concentrations. Moreover, no significant differences or a slight increase of shoot number (**Figure 1C**) (PEG 1% = -0.2, adj-p value = 0.0202; PEG 2% = 0.34, adj-p value = <0.0001; PEG 4% = 1.22, adj-p value = < 0.0001; NaCl 200 mM = 0.85, adj-p value = < 0.0001) and internode length (**Figure 1D**) (PEG 2% = 0.35, adj-p value = 0.0287; PEG 4% = 1.59, adj-p value = < 0.0001; NaCl 200mM = 0.92, adj-p value = < 0.0001) in response to treatment with 50 mM NaCl and 1% PEG, compared to control, was observed. In addition, salt and PEG treatments, compared to control, caused a significant decrease of node number (**Figure 1B**) (PEG 1% = 0.83, adj-p value = < 0.0001; PEG 2% = 1.72, adj-p value = < 0.0001; PEG 4% = 2.58, adj-p value = < 0.0001; NaCl 50 mM = 0.63, adj-p value = 0.0002; NaCl 100 mM = 1.31, adj-p value = < 0.0001; NaCl 200 mM = 2.27, adj-p value = < 0.0001) and shoot length (**Figure 1E**) (PEG 1% = 1.22, adj-p value = < 0.0001; PEG 2% = 1.95, adj-p value = < 0.0001; PEG 4% = 3.5, adj-p value = < 0.0001; NaCl 50 mM = 0.76, adj-p value = < 0.0001; NaCl 100 mM = 1.2, adj-p value = < 0.0001; NaCl 200 mM = 2.99, adj-p value = < 0.0001) among the different concentrations. Taken together, these results suggested a tolerant behavior of cv. Canino to low NaCl, as well as to PEG concentration, leading to a possible adaptation to low severity salt and drought stress.

### 3.2 Effect of drought and salinity on morphological features of olive leaves

**Figure 2:**
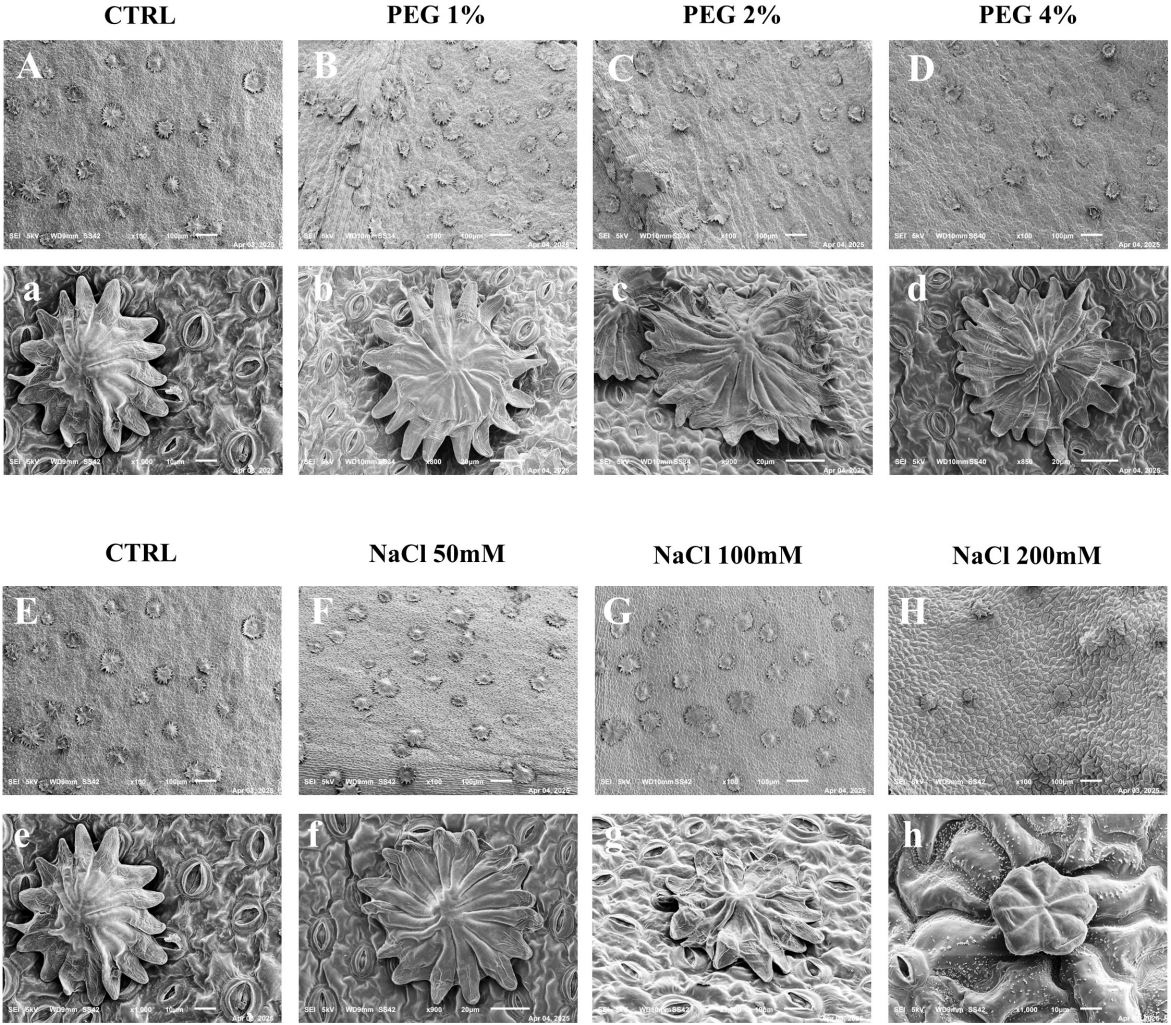
SEM images of olive leaves exposed to PEG (0, 1, 2, and 4%) **(A–D)** and to NaCl treatment (0, 50, 100, and 200 mM) **(E–H)**. Top row: trichome distribution at 100× magnification **(A–H)**. Bottom row: trichome morphology at higher magnifications (900×–1000×) **(a–h)**. Scale bars [**(A–H)**: 100 μm; **(a–h)**: 10 or 20 μm] are indicated on each image.

Scanning electron microscope (SEM) analysis of olive leaves from plants subjected to drought and salinity treatments revealed diverse modifications in stomatal architecture and trichomes (**Figure 2**).

In control samples (**Figures 2A, E**), stomata appeared uniformly distributed and well-formed, although their density was visibly lower than what it is typically observed *in vivo*. This reduction is likely attributed to the controlled environment in which the shoots were grown, characterized by high relative humidity (>90%) and regulated temperature and light intensity. In natural conditions, olive leaves exhibit dense abaxial indumentum and highly heterogeneous trichomes, features that may develop to a lesser extent under constant and non-stressful growth conditions (Fernández et al., 2024). Under PEG treatment, stomata and trichomes showed no evident morphological alterations and distribution across all concentrations tested (**Figures 2B–D, b–d**). Interestingly, the results suggest that PEG, as a non-ionic osmotic agent, imposes water stress without inducing ionic toxicity or causing damage to epidermal structures.

Salinity stress induced concentration-dependent alterations in stomatal and trichome morphology (**Figures 2F–H, f–h**). At 50 mM NaCl, only minimal changes were observed, with stomata maintaining an overall regular structure. At 100 mM NaCl, alterations in stomatal shape and outline became more frequent. At 200 mM NaCl, more pronounced structural modifications were observed. At 200 mM NaCl, several trichomes appeared shrunken or structurally compromised. These changes may reflect ionic or oxidative damage to the cuticle or cell wall components. In *Schizonepeta tenuifolia*, for example, high NaCl concentrations have been shown to increase glandular trichome density while also causing structural collapse of certain trichome types (Zhou et al., 2018), a phenomenon potentially analogous to what observed in this study. Notably, white crystalline-like extrusions were visible on the leaf surface. These may represent salt deposits resulting from active excretion of accumulated ions, a phenomenon observed in other species under severe salinity (Zhou et al., 2018).

### 3.3 Gene expression analysis in olive plants under drought and salt stress

**Figure 3:**
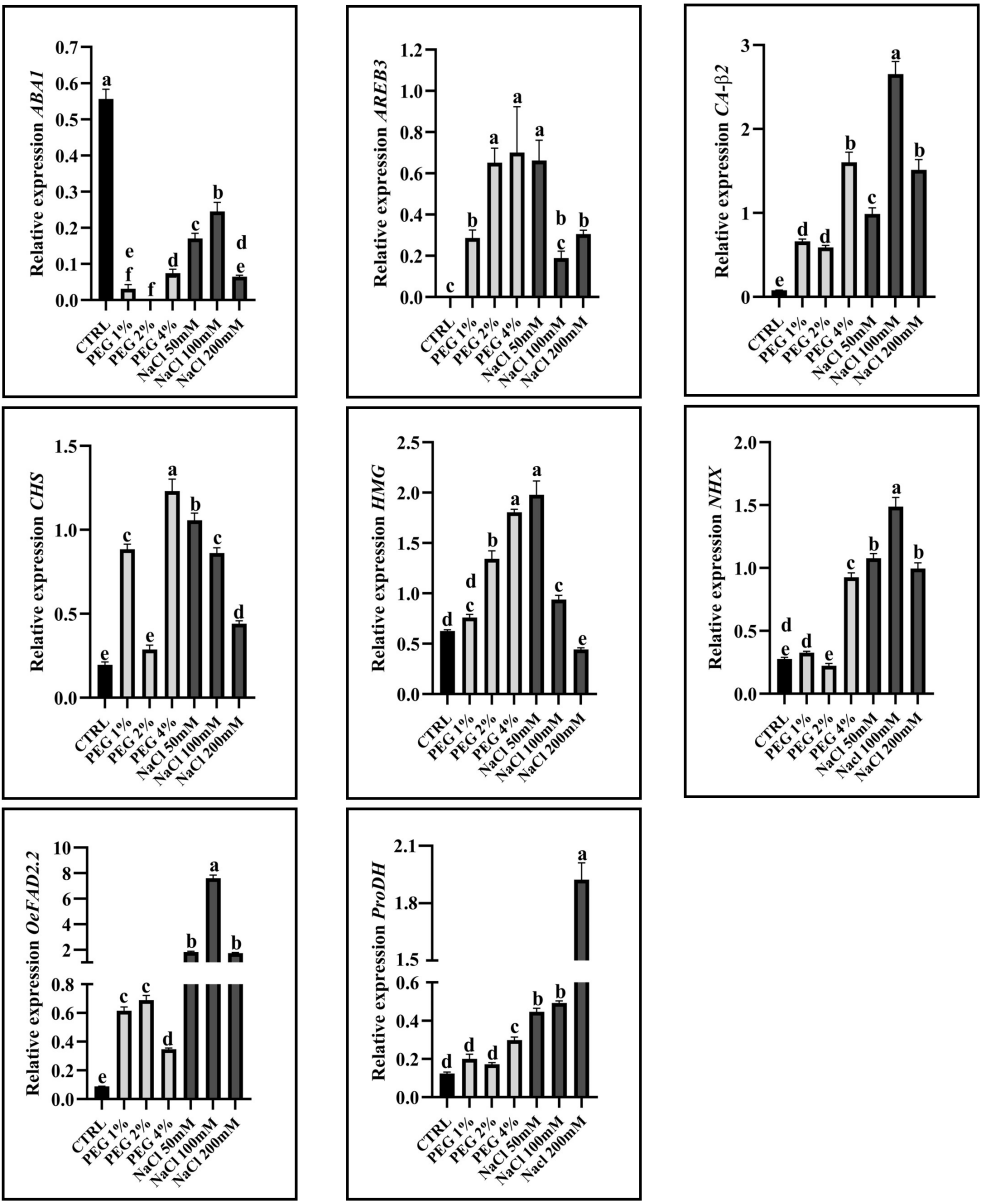
Transcript levels of marker genes relative to the reference gene β-actin after 1%, 2%, 4% PEG and 50 mM, 100 mM, 200 mM NaCl treatments in olive cv. Canino. Zeaxanthin epoxidase (ABA1), ABRE binding protein 3 (AREB3), β2 subunit of carbonic anhydrase (CA-β2), Chalcone synthase (CHS), High mobility group homologous (HMG), Vacuolar Na+/H+ antiporter (NHX), Fatty acid desaturase (OeFAD2-2), Proline dehydrogenase (ProDH). Error bars represent standard deviation. Letters above the bars indicate statistically significant differences among the different conditions (One-way ANOVA; Tukey’s multiple comparison: p-value < 0.05).

In order to identify marker genes responsive to drought and/or salinity stress in cv. Canino, leaves from olive plants subjected to different concentrations of NaCl and PEG were used to carry out a gene expression analysis. In particular, eight genes, found to be differentially expressed under drought and/or salt stress in other olive cultivars (Perez-Martin et al., 2014; Bazakos et al., 2015; Rossi et al., 2016; Moretti et al., 2019; Bashir et al., 2021), were examined in this study to assess if they can be suitable markers also for cv. Canino. Transcript levels of the selected genes are shown in **Figure 3**.

The selected genes are involved in different pathways and mechanisms. Here, PEG and NaCl treatment led to decreasing *ABA1* expression while generally increase the expression of *AREB3*, *CA-β2*, *CHS*, *HMG*, *OeFAD2–2* and *ProDH*, although at different extents. ABA1, also known as ZEP (Zeaxanthin Epoxidase), is a key enzyme in the ABA biosynthetic pathway. In olive plants, as in other species, the role of ABA under drought and salt stress can be significant in regulating water loss, closing stomata and activating stress-responsive genes (Kim et al., 2024; Benny et al., 2020). In our study, the expression of ABA1 decreased under both drought and salt stress, independently of the PEG or NaCl concentration used. This gene epoxidizes the ABA precursors zeaxanthin and antheraxanthin to violaxanthin (Gonzalez-Jorge et al., 2016), thus lower levels of ABA1 could result in a decreased production of violaxanthin and higher levels of zeaxanthin. For this reason, zeaxanthin could be available for photoprotective mechanisms and conversion in caloxanthin and nostoxanthin, involved in the control of plant oxidative stress (Chen et al., 2022). AREB3 belongs to the group of transcription factors known as ABFs (ABRE-Binding Factors) or AREBs; its role in ABA signaling has not been demonstrated as convincingly as the other AREBs/ABFs (Aerts et al., 2024). In olive plants, as in other species, AREB3 is involved in mediating responses to drought and salt stress by regulating the expression of downstream stress-related genes (Benny et al., 2020). Wang et al. (2016a) reported that an overexpression of *AREB3* enhances drought tolerance in *A. thaliana* and increases sensitivity to ABA. Here, *AREB3* was upregulated under any applied concentration of NaCl or PEG, corroborating its impact on drought and salinity response. The β2 subunit of Carbonic Anhydrase (CA-β2) is an isoform of carbonic anhydrase localized in chloroplast membranes (Polishchuk, 2021). CA is a metalloenzyme catalyzing the reversible conversion of CO_2_ and HCO_3_^-^. A higher expression of *CA-β2* is related to the increase in chloroplasts’ conductance, thus improving the CO_2_ usage in photosynthetic processes. Overall, its expression is higher in plants both under salt stress and under drought stress, supporting the hypothesis of CA-β2 involvement in facilitating CO_2_ uptake and utilization for photosynthesis (Perez-Martin et al., 2014; Wang et al., 2016b; Rudenko et al., 2020). Our findings are consistent with previous studies, and the increased expression of *CA-β2* under PEG and NaCl treatment may reflect the need for more efficient CO_2_ usage. Chalcone Synthase (CHS) is a key enzyme involved in the flavonoid biosynthetic pathway, which results in the production of important secondary metabolites in plants with antioxidant activity. In olive plants, CHS plays a significant role in producing compounds that contribute to plant defense, stress tolerance and fruit quality; different olive cultivars may exhibit variation in *CHS* expression and flavonoid accumulation, affecting their stress tolerance and fruit characteristics (Rossi et al., 2016; Hou et al., 2022). In our work, this gene was overexpressed in all treated samples, except for PEG 2% treatment. Interestingly, a similar expression was observed in cv. Frantoio, a salt-tolerant cultivar, under salt stress (Rossi et al., 2016). This data may further support the salt stress tolerance of cv. Canino, as previously observed in Bashir et al., 2021. The High Mobility Group (HMG) homologous proteins are a class of nuclear proteins characterized by several subfamilies with a role in chromatin remodeling activity. Interestingly, it was reported that HMG and two homologous transcription factors, JERF and GRAS, take part of a hierarchical transcription factor network involved in salinity stress response in cv. Kalamon; additionally, an increased expression of the HMG transcript was observed under this stress (Kwak et al., 2007; Bazakos et al., 2015; Xu et al., 2021). Similarly, our findings showed the upregulation of *HMG* in both NaCl and PEG treatments; only in the NaCl 200 mM treated sample *HMG* expression was lower than control. Accordingly, the salt-tolerant cv. Kalamon showed a similar pattern in plant leaves (Bazakos et al., 2015). The Na^+^/H^+^ vacuolar antiporter (NHX) is a membrane protein that plays a crucial role in ion homeostasis, osmotic regulation and stress tolerance in plants, including olive. Its expression is affected by ABA levels, and it is responsible for the exchange of sodium (Na^+^) or potassium (K^+^) ions with hydrogen (H^+^) ions across the vacuolar membrane, which helps to regulate cellular ionic balance and pH under stress conditions (Sodini et al., 2022; Yihong et al., 2024; Muhammad et al., 2025). Rossi et al. (2016) reported an overexpression of *NHX* in the salt-sensitive olive cv. Leccino while no significant differences in the salt-tolerant cv. Frantoio were observed. In our work, *NHX* showed a significant upregulation under severe drought conditions (4% PEG) and following NaCl treatment, despite the salt-tolerant phenotype of cv. Canino, pointing to *NHX* as a potential molecular marker of salt stress in this cultivar. Another gene under study was the Fatty Acid Desaturase 2 (*OeFAD2-2*), that belongs to the FAD genes family which catalyses the desaturation step leading to the conversion of oleic acid to linoleic acid. Among them, the *FAD2–1* and *FAD2–2* isoforms are involved in microsomal linoleic acid production. *FAD2* genes have been isolated and characterized in different plant species (Hernández et al., 2019; Gai et al., 2022; Niu et al., 2022). Moretti et al. (2019) reported that an overexpression of *OeFAD2–2* gene in both drought and salt stresses may help prevent lipid peroxidation and support ion homeostasis by sustaining fatty acid desaturation in the membrane. Notably, the higher expression of *OeFAD2–2* in NaCl-treated samples compared to those treated with PEG may imply a greater capacity of this enzyme to mitigate oxidative stress under salt stress, than under drought conditions. Finally, the last gene analyzed is Proline Dehydrogenase (*ProDH*), an enzyme playing a key role in the metabolism of proline, an amino acid that functions as an osmoprotectant in plants. Specifically, ProDH is an oxidoreductase catalyzing the dehydrogenation of proline in (S)-1-pyrroline-5-carboxylate. In olive plants, ProDH activity was found to be critical for regulating cellular proline levels during and after exposure to environmental stresses such as drought and salinity (Sofo et al., 2004; Hosseinifard et al., 2022; Rudiyanto et al., 2024). Bazakos et al. (2015) reported a higher expression of *ProDH* in olive cv. Kalamon both in roots and leaves under salinity stress condition, indicating the adaptive capacity of this cultivar to abiotic stress. Previous studies on olive cv. Canino under salt stress conditions reported an increased accumulation of proline, a compatible osmolyte playing a key role in maintaining osmotic balance (Bashir et al., 2021). Overall, these results confirm that the analyzed genes, already used in other cultivars as molecular markers for salt and/or drought stress, could also be suitable to assess the response of cv. Canino to the analyzed abiotic stresses.

### 3.4 Role of pigments in energy dissipation and their photoprotective role under drought and salt stress

**Figure 4:**
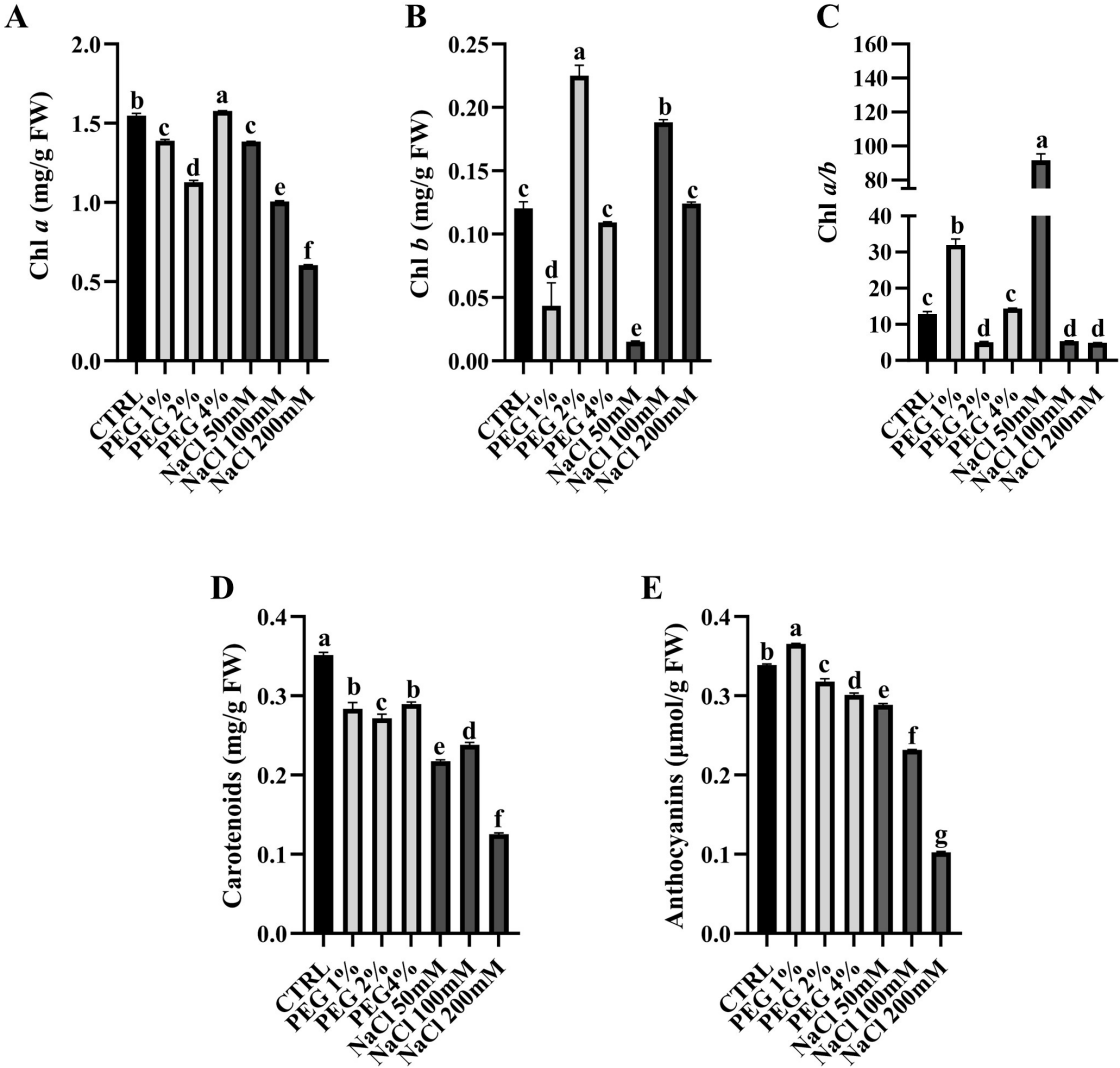
Chlorophylls **(A–C)**, carotenoids **(D)** and anthocyanins **(E)** content in control plants and in PEG- and NaCl-treated plants. Error bars represent standard deviation. Different letters indicate significant differences between treatments (one-way ANOVA; Tukey’s multiple comparison test: p-value < 0.05).

To determine the photosynthetic functionality of plants in response to drought and salt stress, chlorophylls, carotenoids and anthocyanins content was investigated (**Figure 4**).

As reported in **Figure 4A**, Chlorophyll *a* (Chl *a*) content resulted lower in all PEG and NaCl treated samples compared to control, except for 4% PEG. On the other hand, Chlorophyll *b* (Chl *b*) showed a higher expression in 2% PEG and in 100 mM NaCl treated plants (**Figure 4B**). Their ratio (**Figure 4C**), reflecting the balance between light capture and light conversion efficiency, was significantly lower in all samples, except those treated with PEG 1% and NaCl 50 mM. Chl *a* serves as the central molecule for energy transduction in the light-harvesting system and is often downregulated under stress conditions (Falconieri et al., 2024). In contrast, the regulation of Chl *b* in stressed plants is more complex and not thoroughly understood. Its response may vary from that of Chl *a* and across different plant species. Interestingly, several studies have shown that plants may increase Chl *b* levels as a compensatory strategy to help the photosynthetic machinery adapt to stress (Falconieri et al., 2024). In olive trees, drought and salinity stress typically reduce total chlorophyll content due to oxidative damage, reduced photosynthetic activity and inhibited pigment synthesis, although it depends on cultivar resilience and stress intensity (El Yamani and del Pilar Cordovilla, 2024). In fact, drought- and salinity-tolerant cultivars tend to better preserve chlorophyll, contributing to maintaining an appropriate photosynthetic activity under stress (El Yamani and del Pilar Cordovilla, 2024; Muhammad et al., 2024). In our study, the general decrease of Chl *a/b* ratio at higher PEG (2 and 4%) and NaCl (100 and 200 mM) concentrations suggests that plants perceive the stress and try to compensate Chl *a* decrease with Chl *b* increment.

Moreover, to get a better overview of plant response to stress, we also analyzed carotenoids and anthocyanins content (**Figures 4D, E**). Carotenoids, localized mainly in photosystem II (PSII) core, are accessory pigments playing a crucial role in quenching triplet- and singlet-excited chlorophyll to protect photosynthetic tissues by ROS formation (Sharma et al., 2022; Singh and Choudhary, 2022, Michel Havaux, 2023) and prevent photooxidative damage (Singh and Choudhary, 2022). Anthocyanins are a type of flavonoid pigments playing diverse roles beyond being responsible for the color. They act as antioxidants, protect against environmental stressors such as UV radiation and drought, and aid in attracting pollinators and seed dispersers. Furthermore, they can influence plant development and stress responses (Cappellini et al., 2021). In our work all samples showed a decrease of carotenoids and anthocyanins content compared to control under salt stress. Interestingly, in line with our findings, Boussadia et al. (2023) reported a clear reduction of carotenoid content in plants under salt stress. Similarly, Bashir et al., 2021 reported a negative correlation between salt stress and carotenoid level, ascribing these changes to broad metabolic adjustments occurring during plant adaptation to stressful conditions. Furthermore, both carotenoid and anthocyanin content were generally lower than control also in PEG-treated samples. The decrease of these pigments could reflect the influence of a complex regulatory network involving transcriptional control, metabolic reallocation and hormonal crosstalk (Yan et al., 2021). In general, these pigments are often upregulated as a part of the plant defense system, but different studies showed their reduction under moderate and severe drought stress (Nteve et al., 2024; Zheng et al., 2019).

### 3.5 ROS detection in olive cv. Canino in response to drought and salinity stress

**Figure 5:**
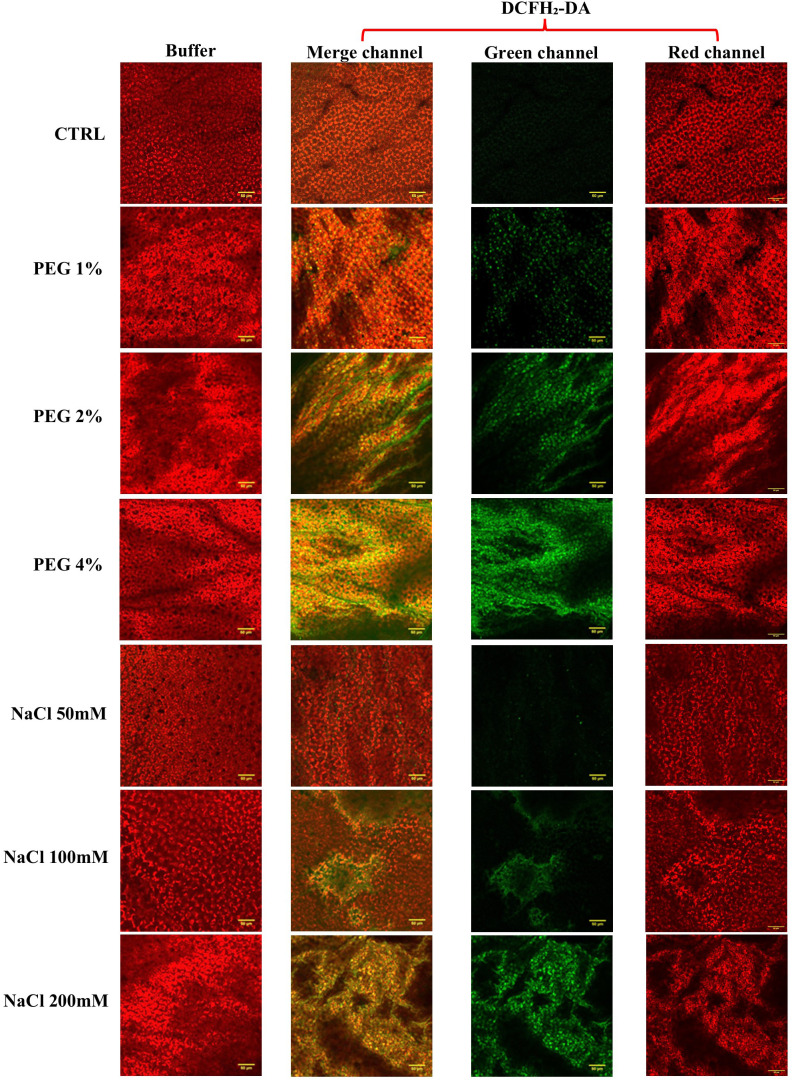
Detection of ROS in PEG and NaCl-treated samples. The detection of ROS was carried out by using 2’,7’-DCFH_2_-DA or buffer (negative technical control). Fluorescence was observed under an LSM 710 confocal microscope with a Plan Neofluar 20/1.30 objective. Two laser excitation lines were used, i.e. 488 for probe detection (green) and 561 nm for chlorophyll auto-fluorescence (red). The bar corresponds to 50 μm. The merged-, green-, and red-channel images are shown. Data was processed using Image J software, version 1.52a.

Increased ROS accumulation under drought or salinity stress has been widely reported in olive and is commonly used as an indicator of oxidative stress during abiotic stress responses (Miller et al., 2010). To evaluate the severity of oxidative stress, reactive oxygen species (ROS) levels were detected in olive plants under PEG and NaCl treatments by using 2,7-dichlorofluorescein diacetate (2,7-DCFH_2_-DA) and confocal microscope observation. DCFH_2_-DA and its derivatives are widely employed as redox-sensitive fluorescent probes for the detection of ROS (Kristiansen et al., 2009). These compounds permeate the plasma membrane and enter the cytoplasm where intracellular esterases cleave their acetate groups, yielding non-fluorescent DCFH_2_. Subsequent oxidation by ROS converts DCFH_2_ to the highly fluorescent (green) 2’,7’-dichlorofluorescein (DCF). While initially believed to exhibit specificity for hydrogen peroxide (H_2_O_2_), recent studies have demonstrated that other ROS, including hydroxyl radicals, lipid hydroperoxides, and peroxynitrite, can also induce oxidation of DCFH_2_, even if with markedly lower efficiency than H_2_O_2_ (Yang et al., 2014). Leaves treated with buffer alone (Tris-HCl 10mM) served as a negative control and showed red fluorescence corresponding to the chlorophyll absorbance spectrum, while samples treated with 2’,7’-DCFH_2_-DA highlighted the presence of ROS, as shown by the green fluorescent signals (**Figure 5**).

**Figure 6:**
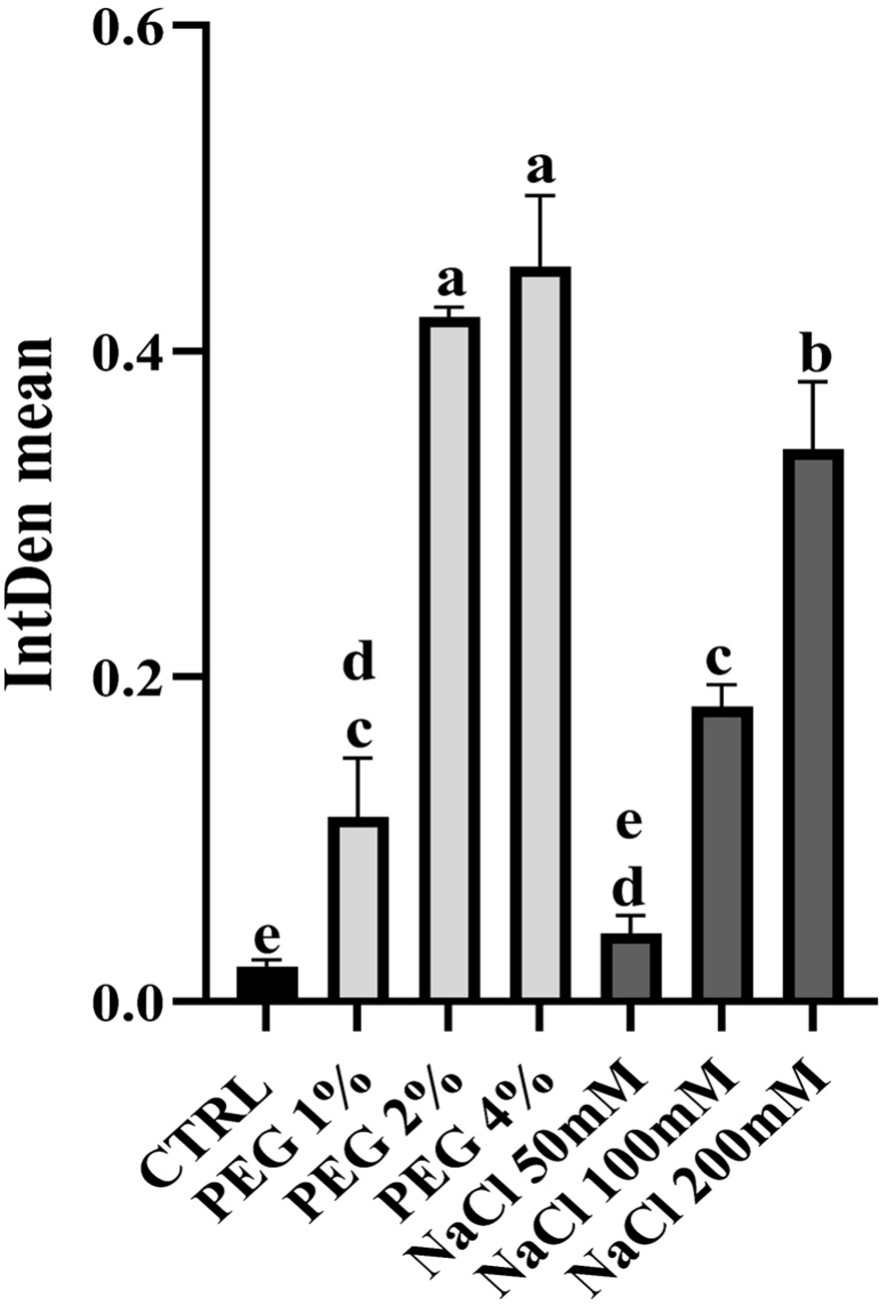
Quantification of green fluorescence detected using DCFH_2_-DA in PEG and NaCl-treated plants. The integrated density mean is reported. Letters above the brackets indicate a statistically significant difference between samples (one-way analysis of variance; Tukey’s test; p-value < 0.05). Quantification has been performed using ImageJ, version 1.52a.

The images revealed that the green fluorescence intensity progressively increased in response to rising drought and salinity stress severity. To corroborate this evidence and to provide a quantitative comparison between samples, green fluorescence intensity was quantified as an integrated density average by using the measurement tools provided by ImageJ software (**Figure 6**).

Interestingly, plants treated with 50 mM NaCl exhibited green fluorescence levels comparable to the control, suggesting that olive cv. Canino exhibits a salt-tolerance phenotype at low NaCl concentration. Nevertheless, treatments with 100 mM and 200 mM NaCl resulted in a marked increase of ROS. This suggested that moderate to severe salt stress may be harmful to olive cv. Canino, in terms of oxidative damage, analogously to what is observed in other cultivars (Palm et al., 2024). Similarly, strong green fluorescence was observed in PEG-treated plants, in particular at 2% and 4% concentrations, confirming elevated ROS level in olive under drought stress (Parri et al., 2024). The heightened oxidative stress observed under both salinity and drought conditions likely contribute to the overall perturbation of plant physiological balance. Due to the pivotal role of oxidative stress in plant stress resilience, we expanded our investigation by exploring lipid peroxidation.

### 3.6 Lipid peroxidation during drought and salinity stress

**Figure 7:**
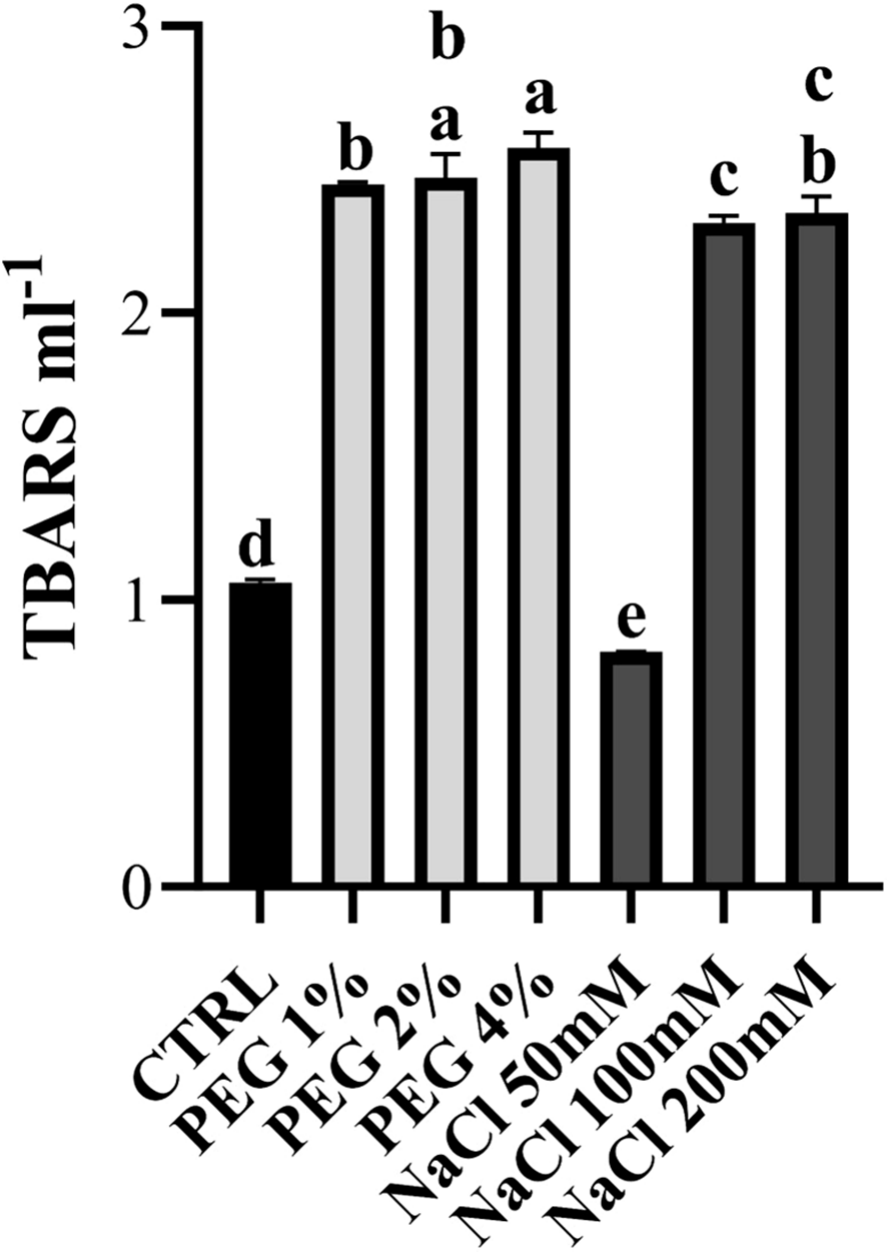
The level of TBARS in olive leaves under 1%, 2%, 4% PEG and 50 mM, 100 mM, 200 mM NaCl is shown. Error bars represent standard deviation. Different letters above the bars indicate statistically significant differences across different conditions (One-way ANOVA; Tukey’s multiple comparison test: p-value < 0.05).

Lipid peroxidation is a process in which free radicals, particularly ROS, attack lipids containing polyunsaturated fatty acids in cellular membranes. This process results in the formation of lipid peroxides and various secondary products that can directly and indirectly alter membrane properties and ion transport capacity, as well as damage protein synthesis processes, eventually resulting in cell death (Proietti et al., 2023). In olive plants, as in other plant species, lipid peroxidation is closely associated with abiotic stresses such as drought, salinity, extreme temperatures and heavy metal exposure (Abdallah et al., 2018; Bashir et al., 2021; Nteve et al., 2024). TBARS content was measured to assess membrane damage caused by increased ROS levels. The TBARS assay allows to measure the malondialdehyde-thiobarbituric acid (MDA-TBA_2_) complex, a spectrophotometrically detectable compound. In our work, PEG-treated samples showed high TBARS levels that were comparable across the three concentrations used. Lipid peroxidation is often reported under water stress (Denaxa et al., 2020). Under salt stress, plants treated with 100 mM and 200 mM NaCl also showed high TBARS levels, while in 50 mM NaCl-treated samples TBARS were lower than the control. This phenomenon could be due to the capacity of mild salt stress to act as a priming stimulus for the plant immune system by activating antioxidant mechanisms that sharpen plant defenses, as observed in *Moringa oleifera* (Azeem et al., 2023) (**Figure 7**). Interestingly, the lipid peroxidation content in salt-treated samples was lower than in PEG-treated ones and this could be linked with the higher *OeFAD2.2* expression observed in the gene expression analysis (**Figure 3**). Indeed, in Arabidopsis *fad2* mutants it has been observed a higher accumulation of lipid peroxidation products (MDA) under salt stress in plants (Zhang et al., 2012). In conclusion, consistent with the ROS detection results, TBARS content reflects increased lipid peroxidation correlated with ROS accumulation.

### 3.7 Pivotal role of enzymatic antioxidant activity in olive plants under drought and salt stress

**Figure 8:**
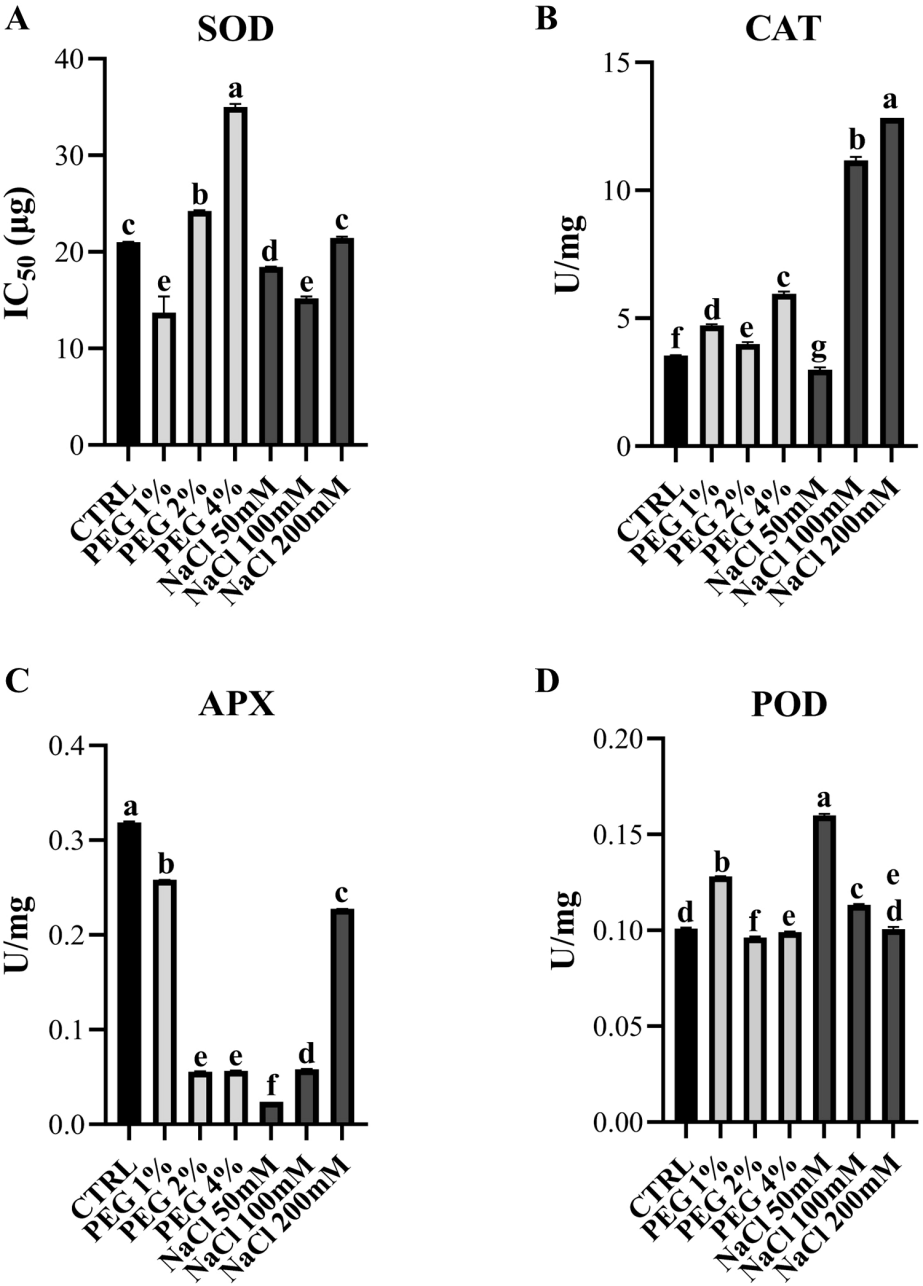
Antioxidant enzymes activities in olive leaves under 1%, 2%, 4% PEG and 50 mM, 100 mM, 200 mM NaCl. **(A)** Superoxide dismutase (SOD), **(B)** Catalase (CAT); **(C)** Ascorbate peroxidase (APX); **(D)** Guaiacol peroxidase (POD). Error bars represent standard deviation. Different letters above the bars indicate statistically significant differences between treatments (one-way ANOVA; Tukey’s multiple comparison test: p-value < 0.05).

ROS are normally produced in both stressed and unstressed cells, acting as signaling molecules. In the latter case, the formation and removal of ROS are well balanced. Nevertheless, when ROS levels increase significantly, the defense system can be overwhelmed. In this condition, plants respond by increasing both enzymatic and non-enzymatic antioxidant processes (Alscher et al., 2002). The enzymatic antioxidant mechanism represents one of the major defense mechanisms against oxidative damage and consists in the activation of antioxidant enzymes, used as an indicator of oxidative stress. Plenty of studies demonstrated the significance of intracellular antioxidant defense machinery against a variety of stresses, including drought and salinity (Xie et al., 2019). The first line of defense against ROS is represented by superoxide dismutase (SOD), belonging to metal-enzyme family that catalyses the dismutation of superoxide anions (O_2_^-^) in molecular oxygen (O_2_) and hydrogen peroxide (H_2_O_2_). Ascorbate peroxidase (APX), catalase (CAT), and guaiacol peroxidase (POD) are among the major H_2_O_2_ scavenging enzymes. CAT detoxifies two molecules of H_2_O_2_ producing H_2_O and O_2_. APX removes H_2_O_2_ working in the ascorbate-glutathione cycle (Omoarelojie et al., 2021). Finally, POD produces tetraguaiacol molecules using H_2_O_2_ (Abboud et al., 2021). In this work, we analyzed the activity of SOD, CAT, APX, POD in olive leaves under stress. Noteworthy, the activity of SOD is reported as IC_50_ which represents the percentage of inhibition of formazan formation. Therefore, a higher IC_50_ value corresponds to lower SOD activity in the protein extract used in the assay. As shown in **Figure 8A**, while SOD activity is generally higher in all plants under NaCl treatment compared to control, plants under moderate to severe drought stress exhibited lower SOD activity, which probably could be one of the reasons of the higher oxidative stress observed in PEG treated plants.

As shown in **Figure 8B**, both drought and salinity stress generally induced CAT activity, except for samples treated with 50mM NaCl, likely due to the salt-tolerance behavior of olive cv. Canino at low salt concentration. This result corroborates the lower ROS production after treatment with 50 mM NaCl (**Figure 5**). All stressed samples revealed a lower activity of APX compared to control (**Figure 8C**), suggesting that H_2_O_2_ is preferably detoxified via CAT activity. Finally, in all samples under moderate and severe stress conditions POD activity drops slightly compared to control (**Figure 8D**). In contrast, plants treated with 1% PEG and 50 mM NaCl exhibited a notable increase in enzyme activity, suggesting an additional defense strategy aimed at preventing excessive ROS accumulation. In general, as shown in **Figure 6**, plants treated with NaCl showed a lower oxidative stress compared to those treated with PEG. This could be due to the enhanced activity of SOD, CAT and POD in NaCl-treated samples, while plants under drought stress showed reduced activity of these antioxidant enzymes. This finding supports the hypothesis that cv. Canino may struggle to maintain sufficient antioxidant enzymes activity under drought stress. These results align with those from ROS detection (**Figures 5**, **6**) and lipid peroxidation assays (**Figure 7**). Overall, our study highlights a pattern of antioxidant enzymes activity in cv. Canino that is consistent with that observed in other cultivars (Regni et al., 2019; Gholami et al., 2022).

## 4 Conclusions

The results of the present work contributed to disclose the molecular bases of defense responses in olive (*Olea europaea* L.) cv. Canino under drought and salinity stress, two of the principal abiotic factors affecting olive cultivation in the Mediterranean basin. The qRT-PCR analysis allowed to identify a total of eight genes (*ABA1*, *AREB3*, *CA-β2*, *CHS*, *HMG*, *NHX*, *OeFAD2.2* and *ProDH*) that can be used as molecular markers for drought and/or salinity stress in cv. Canino. Furthermore, by examining photosynthetic pigments and carotenoids and anthocyanins levels, it was possible to gain a deeper understanding of the mechanisms involved in energy accumulation and conversion in cv. Canino under stressful conditions. These analysis revealed a reduction of photosynthetic efficiency and pigment concentration associated with increasing concentration of NaCl and PEG. Lipid peroxidation and ROS assays showed a higher oxidative stress in plants treated with PEG compared to those treated with NaCl, that could be consistent with the salt-tolerance behavior of cv. Canino, as previously observed (Bashir et al., 2021). However, we can hypothesize that the mild responses observed at 50 mM NaCl could suggest two possible interpretations: a priming effect, in which defense mechanisms are activated without causing significant damage, or the intrinsic tolerance of this cultivar to low salinity. Distinguishing between these scenarios requires comparative studies across multiple olive genotypes and a broader range of salinity levels, ideally combining physiological and molecular markers of stress activation. Overall, our results provide a good benchmark for further investigations aimed at unraveling molecular mechanisms underlying plant responses to abiotic stress in cv. Canino, possibly on *in vivo*-grown plants. Noteworthy, our findings provided detailed insights into the morphological, physiological, and molecular responses of cv. Canino to drought and salinity stress, and further comparative studies involving multiple olive cultivars would be beneficial to determine whether these responses are conserved or cultivar-specific.

## Data Availability

The datasets presented in this study can be found in online repositories. The names of the repository/repositories and accession number(s) can be found below: https://doi.org/10.5281/zenodo.18984977, ID: 18984977.

## Supplementary material

Supplementary Table 1 RNA concentration estimated for all samples.

Supplementary Table 2 List of primers used in the study for target/reference genes.

Supplementary Figure 1 Representative photos of *in vitro*-grown olive plants.
