# Supplementary figures and images for: Molecular and morphological signatures of drought and salinity stress in Olea europaea
Source: Front Plant Sci. 2026 Apr 14;17:1813434. doi: 10.3389/fpls.2026.1813434 (PMC13125883; doi:10.3389/fpls.2026.1813434)

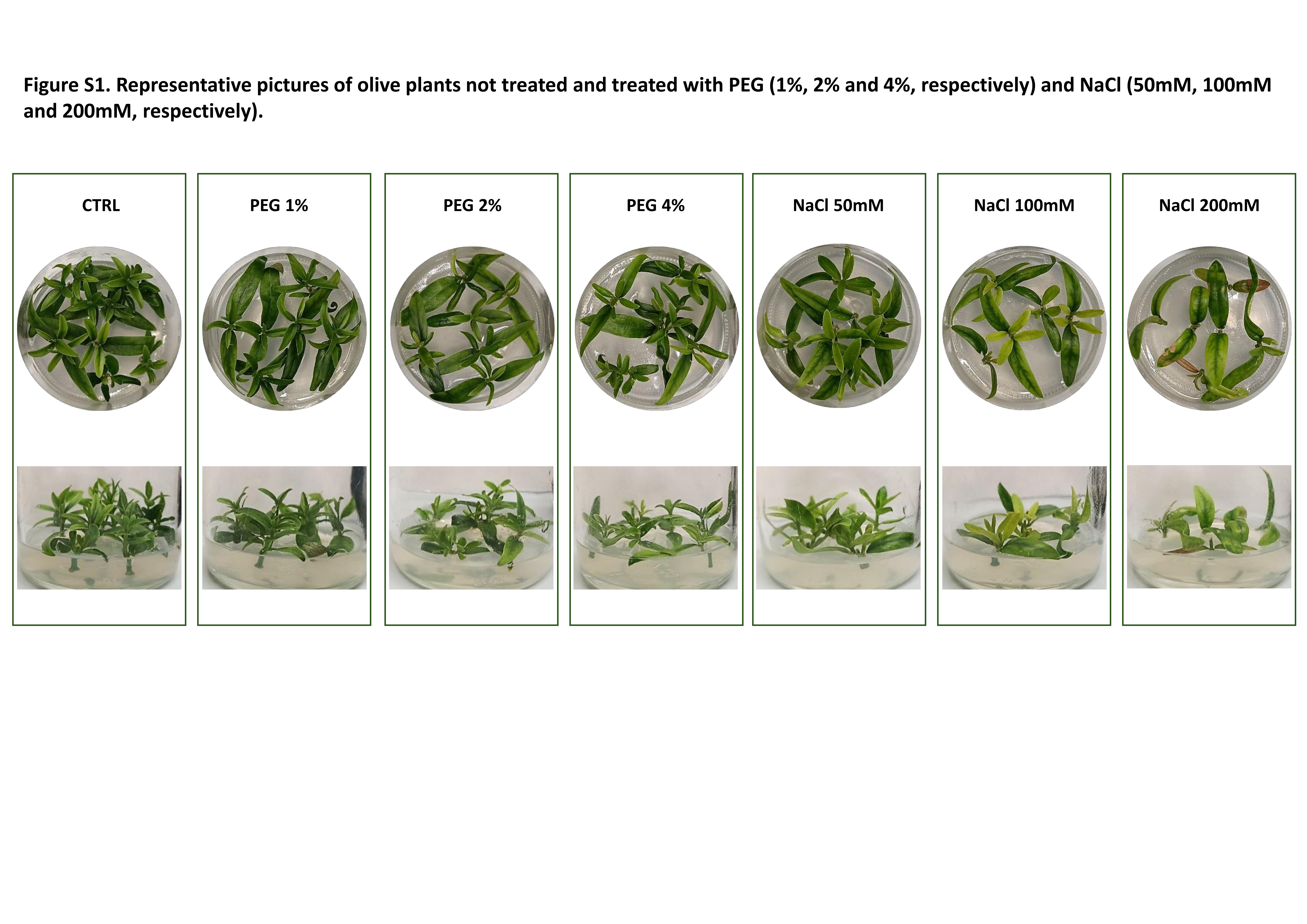

Supplement: Supplementary Table 1 — RNA concentration estimated for all samples. [file Image1.jpeg]
