# Supplementary material for: Molecular and morphological signatures of drought and salinity stress in Olea europaea
Source: Front Plant Sci. 2026 Apr 14;17:1813434. doi: 10.3389/fpls.2026.1813434 (PMC13125883; doi:10.3389/fpls.2026.1813434)
Supplement: Supplementary Table 2 — List of primers used in the study for target/reference genes. [file Table1.docx]

**Table S1. RNA concentration of each sample.**

| **Condition** | **Rep** | **Concentration (μg/μL)** |
| --- | --- | --- |
| Ctrl | 1 | 0,7 |
| Ctrl | 2 | 1,1 |
| Ctrl | 3 | 0,57 |
| PEG 1% | 1 | 0,52 |
| PEG 1% | 2 | 0,51 |
| PEG 1% | 3 | 0,48 |
| PEG 2% | 1 | 0,37 |
| PEG 2% | 2 | 0,64 |
| PEG 2% | 3 | 0,46 |
| PEG 4% | 1 | 0,76 |
| PEG 4% | 2 | 0,54 |
| PEG 4% | 3 | 0,44 |
| NaCl 50mM | 1 | 0,46 |
| NaCl 50mM | 2 | 0,57 |
| NaCl 50mM | 3 | 0,47 |
| NaCl 100mM | 1 | 0,5 |
| NaCl 100mM | 2 | 0,23 |
| NaCl 100mM | 3 | 0,57 |
| NaCl 200mM | 1 | 0,49 |
| NaCl 200mM | 2 | 0,63 |
| NaCl 200mM | 3 | 0,26 |
