# Supplementary material for: Molecular and morphological signatures of drought and salinity stress in Olea europaea
Source: Front Plant Sci. 2026 Apr 14;17:1813434. doi: 10.3389/fpls.2026.1813434 (PMC13125883; doi:10.3389/fpls.2026.1813434)
Supplement: Supplementary Figure 1 — Representative photos of in vitro-grown olive plants. [file Table2.docx]

**Table S2.** List of primers used in the study for target/reference genes.

| **Genes** | **Acc. number/Genbank** | **Primer sequences (Forward/Reverse)** | **Reference** |
| --- | --- | --- | --- |
| β-Act_F | AF545569.1 | ACTATGAACAGGATCTTGAG | Rossi *et al.*, 2016 |
| β-Act R | AF545569.1 | GAACCACCACTGAGGACGAT | Rossi *et al*., 2016 |
| AREB3_F | AF545569.1 | TCTGATTGCTACAACGCCGT | Designed |
| AREB3 R | AF545569.1 | GAGAGTGGCGAAACATGCAGA | Designed |
| CA-β2 F | AT5G14740 | ATATGCAGGAGTTGGAGCCG | Designed |
| CA-β2_R | AT5G14740 | CATAAGCCCCTTGATGCCACC | Designed |
| CHS F | AF384049.1 | ACCGGATGACACTCATTTGGA | Rossi *et al*., 2016 |
| CHS_R | AF384049.1 | GCGCCGTCGCCAAAC | Rossi *et al*., 2016 |
| HMG F | GRNLHQF09FN48K | CAAGTCCAAAATCCGAAGTGAATG | Bazakos *et al*., 2015 |
| HMG R | GRNLHQF09FN48K | CTCTCTACTCGTCGTCGTCATCT | Bazakos *et al*., 2015 |
| NHX_F | EF506515.1 | CGTCAAATACCACCGAAGCA | Rossi *et al*., 2016 |
| NHX R | EF506515.1 | GCCATAGCCCCTCGAAGTC | Rossi *et al.*, 2016 |
| OeFAD2-2_F | Y733077.1 | CTTGTGGGCTTTACCGTCTC | Moretti *et al.*, 2019 |
| OeFAD2-2 R | Y733077.1 | AGGGAGGGATGTGTATGCTG | Moretti *et al.*, 2019 |
| ProDH F | GRNLHQF11GW49N | GGCCTATTCAGGGAGTGGTAAA | Bazakos *et al*., 2015 |
| ProDH R | GRNLHQF11GW49N | CTACTGAGATGGCAATACAAGGAT | Bazakos *et al*., 2015 |
| ZEP F | NM 180954.3 | TCAACCTTGCATCGTCGGAAGG | Designed |
| ZEP R | NM 180954.3 | AACACACGAGCATGCATCTTCG | Designed |
